# Authentication and Provenance of Walnut Combining Fourier Transform Mid-Infrared Spectroscopy with Machine Learning Algorithms

**DOI:** 10.3390/molecules25214987

**Published:** 2020-10-28

**Authors:** Hongyan Zhu, Jun-Li Xu

**Affiliations:** 1 College of Electronic Engineering, Guangxi Normal University, Guilin 541004, China; hyzhu-zju@foxmail.com; 2 UCD School of Biosystems and Food Engineering, University College of Dublin (UCD), Belfield Dublin 4, Ireland

**Keywords:** walnut, Fourier transform mid-infrared spectroscopy, successive projection algorithm, genetic algorithm-partial least squares, machine learning

## Abstract

Different varieties and geographical origins of walnut usually lead to different nutritional values, contributing to a big difference in the final price. The conventional analytical techniques have some unavoidable limitations, e.g., chemical analysis is usually time-expensive and labor-intensive. Therefore, this work aims to apply Fourier transform mid-infrared spectroscopy coupled with machine learning algorithms for the rapid and accurate classification of walnut species that originated from ten varieties produced from four provinces. Three types of models were developed by using five machine learning classifiers to (1) differentiate four geographical origins; (2) identify varieties produced from the same origin; and (3) classify all 10 varieties from four origins. Prior to modeling, the wavelet transform algorithm was used to smooth and denoise the spectrum. The results showed that the identification of varieties under the same origin performed the best (i.e., accuracy = 100% for some origins), followed by the classification of four different origins (i.e., accuracy = 96.97%), while the discrimination of all 10 varieties is the least desirable (i.e., accuracy = 87.88%). Our results implicated that using the full spectral range of 700–4350 cm^−1^ is inferior to using the subsets of the optimal spectral variables for some classifiers. Additionally, it is demonstrated that back propagation neural network (BPNN) delivered the best model performance, while random forests (RF) produced the worst outcome. Hence, this work showed that the authentication and provenance of walnut can be realized effectively based on Fourier transform mid-infrared spectroscopy combined with machine learning algorithms.

## 1. Introduction

Walnut is the hard-shell fruit, which is well-known for its high nutritional value [1]. The walnut kernel is rich in protein, fatty acids, a variety of trace elements, and other nutrients beneficial to the human body [2]. The walnut kernel contains a high amount of oil by weight, ranging from 52% to 70% depending on the environmental conditions, cultivars, and geographic location [3]. Researchers [4] reported that a walnut-enriched diet had a beneficial effect on cardioprotection and bone loss. Since walnut fruit is found to be very rich in phenolic compounds, it exhibits a wide spectrum of biological activities, e.g., anti-inflammatory, antioxidant, and antitumor properties [5]. Walnut also plays an important role in medication, and it has been used for different medical purposes in China and Europe [2,6]. The walnut belongs to the Juglandaceae family that contains six genera, with the most important two being *Juglans* and *Pterocarya*. Persian walnut (*Juglans regia* L.), which is widely cultivated in Asia (with China as the top producer), the U.S., and Europe, has the highest quality among the walnut varieties. It is well recognized as a sweet taste, a relatively large kernel, and a thin shell, which makes it easy to crack [7]. Generally, the composition of walnut varies with geographic origins, thanks to a range of regional differences in soil, climate, and agricultural practice, as well as the different subvarieties that are involved (i.e., different cultivars are apt to be grown in different regions) [8]. Since the quality and nutritional value of a walnut highly depend on its geographic origin and variety, it is of great importance to develop new and increasingly sophisticated techniques for the authentication and provenance of walnut, which is desirable for agricultural farmers, retailers, consumers, and administrative authorities [9].

The use of geographical indications allows producers to obtain market recognition and often a premium price. The false use of geographical indications by unauthorized parties is detrimental to consumers and legitimate producers [9]. Considering geographic specifications of quality, numerous studies have been conducted to classify food products based on their geographical locations [10]. Although it is often viewed as a consumer issue, the major drive for appropriate analytical methods to confirm authenticity has come from the food processing industry and regulatory bodies. The most available research studies are based on the measurement of certain chemicals, such as the fatty acids and multi-element composition [11], which is normally obtained by using a broad variety of instrumental techniques, e.g., gas chromatography with mass spectrometry (GC-MS) [12] and nuclear magnetic resonance (NMR) [13]. To discriminate the cultivars and geographical origins of walnut, current studies focus on the analysis of the major and minor compounds, such as volatiles, fatty acids, polyphenols, sterols, and minerals [14] or assessment of the antioxidant, oxidative stability, and antimicrobial activity [15]. The method to determine chemical components is usually laborious and time-consuming, requiring complex sample preparation. Under this circumstance, an alternative method, which enables delivering a rapid and accurate result, is highly desired.

Infrared spectroscopy, including near infrared (NIR) and mid-infrared (MIR), has been widely researched in the identification and constituent analysis of food products in both qualitative and quantitative manners [16,17,18,19]. Both techniques are rapid, straightforward, and sensitive with moderate instrument cost and relative ease of sample presentation, showing great potential in the field of food quality control analysis. The broad and overlapping spectral peaks in NIR make it difficult for spectral interpretation and sometimes quantitative analysis, although NIR light facilitates a high penetration depth compared to MIR [20]. MIR absorption peaks are easy to assign to different functional groups due to the fundamental vibrations of molecular bonds. Therefore, MIR, which produces well-resolved absorption bands, could be more associated with some types of compounds in food under investigation [21]. MIR spectroscopy has found numerous applications in food quality analysis [22,23]. For instance, a recent study [24] was carried out to apply Fourier transform mid-infrared (FT-MIR) spectroscopy for the rapid nutritional profiling of pea seeds. Their work produced the correlation coefficients greater than 0.83 for the prediction of protein, fiber, and phytic acid concentrations in seed, suggesting the novelty and usefulness of FT-MIR as a simple, fast, and cost-effective technique to determine multiple seed constituents simultaneously. Furthermore, MIR methods have been reported for the authentication, provenance, and traceability of various food products, e.g., fruit purees [25], honey [26], and cocoa bean shell [27]. Recently, Formosa et al. [28] applied attenuated total reflection mid-infrared (ATR-FT-MIR) spectroscopy in discriminating and classifying local honey from that of foreign origin. A high accuracy (>95%) was achieved by using different modeling algorithms with spectral pre-treatments, confirming the capability of MIR in the context of the authentication of honey samples.

To the best of knowledge, this study is the first to investigate the performance of FT-MIR combined with different machine learning algorithms to classify 10 varieties of walnut. This study also attempts to apply spectral pre-treatment to denoise the spectrum prior to modeling. In addition, different subsets of optimal spectral variables will be created by using uninformative variable elimination (UVE) combining with successive projection algorithm (SPA) and genetic algorithm–partial least squares (GA-PLS), and their performance will be compared against using the entire spectral range. Different machine learning classifiers will be compared in terms of (1) classifying geographic origins, (2) classifying varieties under the same geographic origins, and (3) classifying all 10 varieties.

## 2. Results

### 2.1. Spectral Profiles and Pre-Treatment

**Figure 1:**
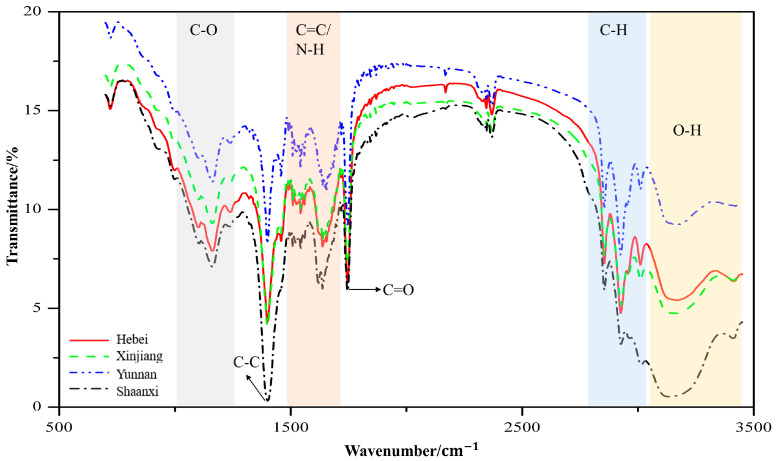
The preprocessed mean spectra calculated from each geographic origin.

The broad variability in mean FT-MIR spectra obtained among four geographic origins is presented in Figure 1. The broad absorption band in 3000–3500 cm^−1^ is indicative of the existence of a hydroxyl group attributed to the stretching vibration of an O–H. A series of peaks existing in 2800–3000 cm^−1^ correspond to the C–H stretching vibration of alkane [29]. The sharp band at 1740 cm^−1^ is attributed to the C=O stretching of the carbonyl group, while the bands observed at 1500–1700 cm^−1^ may either be attributed to the C=C stretching of alkene or N–H bending of amine [30]. A notable band located at 1400–1500 cm^−1^ may be assigned to the C–C stretching of the aromatic ring [31]. Numerous bands appearing in 1000–1275 cm^−1^ might originate from C–O stretching [32]. Figure 1 displays distinguishable differences in terms of band shapes and areas among the mean spectra collected from different origins, suggesting that FT-MIR is capable of capturing specific characteristics of walnut products that are influenced by geographically specific factors.

### 2.2. Optimal Wavenumbers Selection

Herein, two variable selection strategies, namely, UVE-SPA and GA-PLS, were adopted to select the subsets of important spectral variables from the full range originally consisting of 2853 variables.

**Figure 2:**
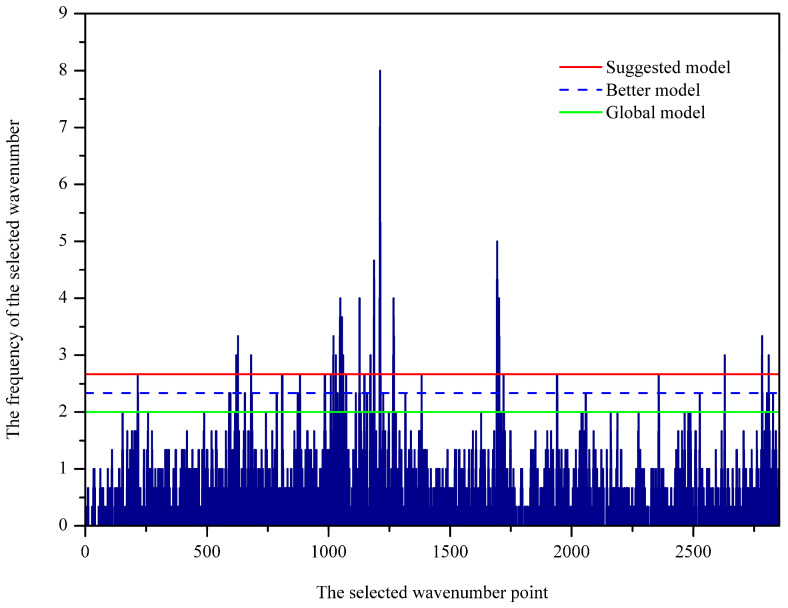
The histogram of frequency to be selected for individual variables.

**Figure 3:**
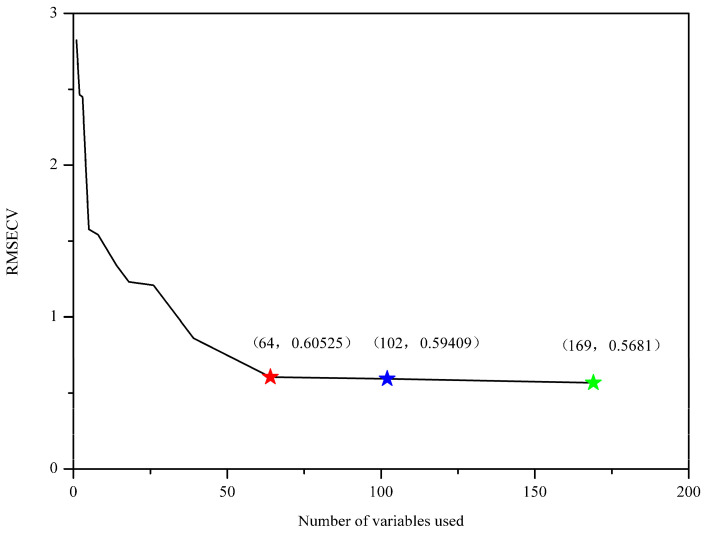
The root mean square error of cross-validation (RMSECV) of the number of variables included. The global, the better, and the suggested model are marked with green, blue, and red stars, respectively.

Sorting the importance of variables is crucial for variable selection and model simplification. GA-PLS seeks for the importance of variables based on the frequency of selection. As shown in Figure 2, the frequency of the selected wavenumbers was indicated. In general, variables with relatively large frequency are more important to the classification modeling and therefore should be chosen. In GA-PLS, the variance coefficients (CV) and the root mean square error of cross-validation (RMSECV) were computed from the subset of the selected variables. Figure 3 displays the evolution of RMSECV against the number of selected variables, while CV is plotted against the number of selected variables and shown in Figure S4. As seen, the global, the better, and the suggested model are marked with green, blue, and red stars, respectively. It is noted that RMSECV decreases rapidly at the first 64 selected variables and then tends to slow down, meaning that the increase of more variables makes little contribution to the model performance. For this reason, 102 important wavenumbers were selected and shown in Figure S5, which was based on the better model in the GA-PLS.

**Figure 4:**
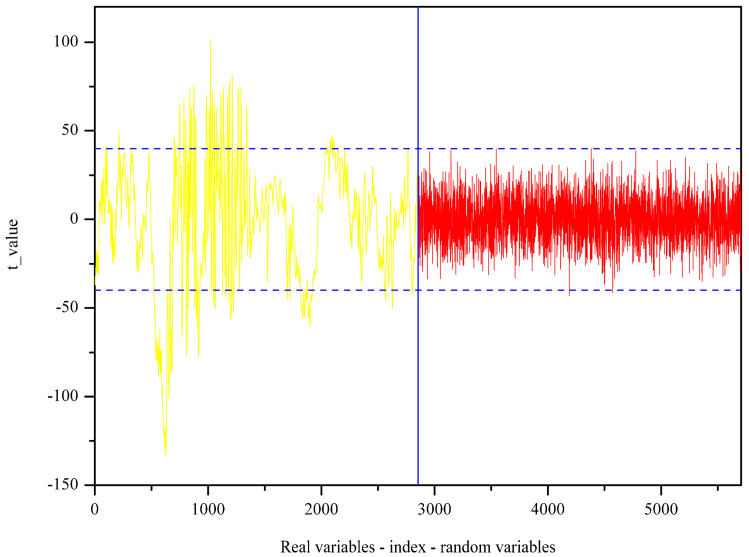
The stability of individual variables obtained by applying uninformative variable elimination (UVE).

**Figure 5:**
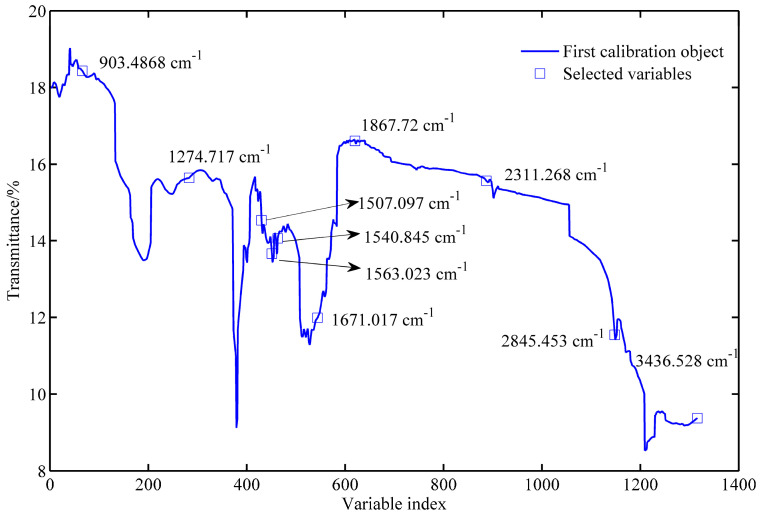
The selected spectral variables after performing UVE-SPA (uninformative variable elimination combining with successive projection algorithm).

In the UVE process, the PLS algorithm obtained the optimal principal factor of 12. Figure 4 shows the stability of each variable after UVE modeling. The left part of the vertical line in yellow was the spectral wavenumber variables, and the right part shown in a red color was random variables. Two horizontal lines represent the minimum and maximum cutoff lines. The stability of the wavenumbers in the middle of the two lines meant that the spectral information carried by the wavenumbers was useless. By this means, 1316 wavenumber variables were determined as important and therefore selected and fed into SPA as input. The significant variables are selected based on the minimum root mean square error (RMSE) in SPA. Figure S6 exhibits the change of RMSE along with the selected variable increases from 1 to 35. As seen, 10 spectral variables have an impact on reducing RMSE, and therefore, they are chosen. The selected variables are subsequently shown in Figure 5.

### 2.3. PCA Exploration

**Figure 6:**
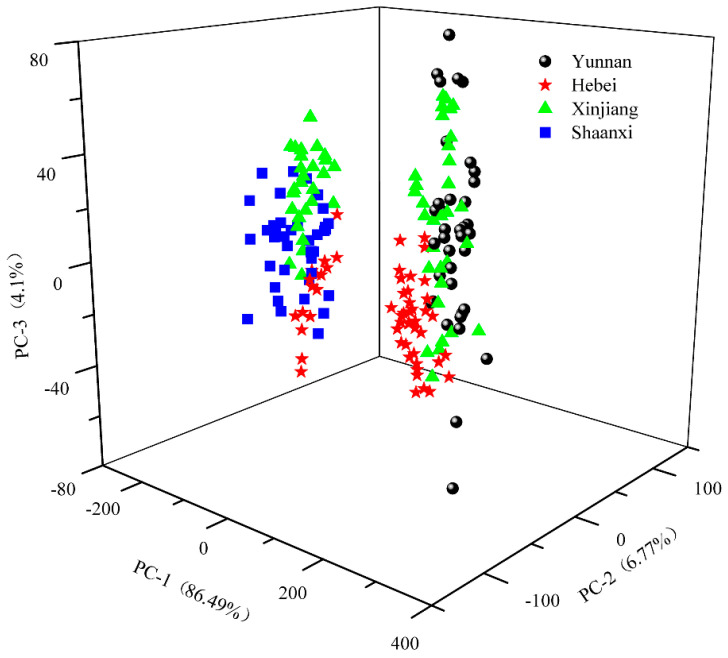
The score plot of the first three principal components (PCs) with different geographic origins highlighted in different markers.

PCA, developed from the pre-treated spectra data, was used to explore the dataset before the classification model establishment. Figure 6 demonstrates the score plots for samples collected from four geographic origins with PC1 explaining 86.49% of variation, PC2 explaining 6.77%, and PC3 explaining 4.1%, accounting for 97.36% of the variance represented by the first three PCs. The figure shows samples collected from Shaanxi province all located at the negative side of PC1. In contrast, samples of Yunnan are all found to have positive scores in PC1. This indicates that walnut samples harvested from Yunnan and Shaanxi provinces are distinctly different. Yunnan has a generally mild climate with pleasant and fair weather because of the province’s location on south-facing mountain slopes, receiving the influence of both the Pacific and Indian oceans. However, Shaanxi has a continental climate with a cold winter and hot summer. In this sense, the difference in the growth environment (temperature, humidity, rainfall, light time, etc.) could contribute to distinctive characteristics in samples from these provinces, which is consistent with published research on olive oils from different geographic origins displaying various quality attributes [33,34]. As for samples from Hebei and Xinjiang provinces, it is interesting to find that some of them are distributed at a negative axis and some are a positive size, suggesting the variability within the same origin probably due to different varieties. In addition, PCA score plots, created from each geographic origin, are presented in Figure S7 of the supplementary material. The results indicate that two varieties collected from the same provinces (i.e., Yunnan and Shaanxi) formed separable clusters. To visualize the possibility of classifying all 10 varieties, the same score plot of Figure 6 is modified to highlight each variety, as shown in Figure S8. It is seen that some varieties are heavily overlapping with the others, making a challenging job for discrimination.

### 2.4. Classification of Geographic Origins

Classification models are developed from the entire spectral region as well as the selected variables, and the results are summarized in Table 1. It should be noted that the model performance is evaluated and compared based on the external test set. Although different classifiers produce dissimilar model performances, the classification of four geographic origins is relatively satisfying with the average accuracy higher than 80%. It is probably because the quality of the walnut varies depending on the geographic origins due to different growth environments. Similar results can be found in the literature. For example, Vermeulen et al. [35] applied attenuated total reflection-Fourier transform infrared (ATR-FTIR) to the oil fraction extracted from the dried distillers grains with solubles for classification of origins. The model developed from their study provided a classification accuracy higher than 95% using an external validation set.

It is also noted that different classifiers respond differently with regard to the comparison between the full spectral range and the selected variables. A better predictive ability is witnessed after removing unimportant variables for ELM and PLS-DA classifiers. Interestingly, RF produced the same overall accuracy over the different spectral subsets, i.e., full, which was selected by UVE-SPA and selected by GA-PLS. The inferior model performance of RF suggests that this classifier is unsuitable for walnut powder discrimination based on FTIR spectra. However, RBF neural network works better on the full spectral region compared the reduced subsets. The model performances between these two variable selection methods are dissimilar, which is expected due to the different mathematical computations in recognizing important wavenumbers. Table 1 shows that the best result is achieved by using BPNN built from the selected variables with the overall accuracy higher than 95%.

### 2.5. Classification of Varieties Under the Same Origin

The next step is to investigate if the classifiers can distinguish different varieties under the same geographic origin. In this sense, classification models for the identification of varieties are separately developed within the same origin, as shown in Table 2. Compared to the discrimination of four origins (see Table 1), a better classification performance is evidenced. It is noticed that the classification of varieties in Yunnan and Shaanxi provinces outperformed those in Xinjiang and Hebei, which is in line with the PCA score plots in the Supplementary Materials. Overall, the models built from the selected variables are superior to those from the full spectral region. The results based on the optimal wavenumbers selected by GA are close to the results of using the UVE-SPA algorithm. Again, BP neural network is seen as the most powerful algorithm that produces the best classification performance almost in all cases, while RF is recognized as the least desirable classifier, generating the worst accuracy in a test set.

### 2.6. Classification of All Varieties

Classification of all 10 varieties coming from four geographic origins are subsequently explored, with the statistical results summarized in Table 3. The inferior classification performance is observed when compared to the discrimination of origins or varieties (Table 1) within the same origin (Table 2). This is understandable, since the developed model is more complicated with more classes, e.g., 10 varieties vs. four origins. This also reminds us of the PCA score plots in which samples of different varieties are not visually clustered. As the most powerful classifier, BPNN reaches the accuracy of 87.88% and 83.33% using the subset of variables selected from GA-PLS and UVE-SPA, respectively.

## 3. Discussion

The interaction of mid-IR radiation with a walnut sample provides a spectral fingerprint useful for discrimination. FT-MIR enables recording spectral characteristics of walnut products related to the four geographic origins. Good separation between four origins is noticed in the mean FT-MIR spectra of Figure 1, suggesting the powerfulness and capability of vibrational spectroscopy for the discrimination of walnut’s geographical origin. Due to the diversity of walnut samples within the same geographic origin, machine learning algorithms are required. Generally, machine learning algorithms are capable of addressing some random noise with a large size of training samples. In our case, since the training set is relatively small, the random noise is likely to cause overfitting problems; i.e., the model wrongly uses noise as a feature and performs well on the training set, yet it performs poorly on the test set. For this reason, the wavelet transform algorithm is a good option for spectral smoothing prior to machine learning model development.

Both the unsupervised (i.e., PCA) and supervised machine learning methods evidenced the presence of differences between the walnuts having diverse provenance. Such differences are unlikely due to random variation or overfitting issues. A PCA score plot (see Figure 6) exhibits that walnuts harvested from Shaanxi province can be well separated from samples from Yunnan. Since the main factor under investigation is the geographical origin, it is reasonable to ascribe the samples separation to the distinct environmental features of these two provinces. Variations of soil and weather conditions are likely to influence the walnut’s chemical composition.

As pointed out by Table 1, Table 2 and Table 3, the selection of important variables has the potential to increase the model performance for some classifiers. Results also show that the model performance varies distinctively between ML algorithms. It is understandable, since each machine learning algorithm works in different manners and they have been designed for various applications. Overall, the results proved that BPNN performed the best at all conditions, suggesting the suitability of applying BPNN on FTIR spectral data. On the contrary, RF presented the worst performance, and therefore, it is not suggested to apply RF for such applications. RBF modeling was able to produce good results, yet it was unstable in some circumstances. The discrimination of varieties within the same origin performed the best, yet this mode requires more classification models (i.e., one model for each origin). The classification of geographic origins is also desirable, with the highest accuracy of 96.97% achieved by UVE-SPA-BPNN. The identification of all 10 varieties in one attempt is the least satisfying with the highest accuracy of 87.88% by GA-PLS-BPNN. Therefore, it is recommended to classify the geographic origins first and then apply different models to identify varieties under the same origin.

In spite of the remarkable outcomes, we feel obliged to point out that the development of a comprehensive model that is able to discriminate the geographic origin of an unseen walnut sample is not feasible at the moment. An exhaustive sampling of world walnut samples over several harvest years is required. The future work will include more walnut samples with a large variability. For example, seasonal climate fluctuations will be taken into consideration by repeating the sampling over several consecutive harvesting seasons. This extra variation is beneficial to ensure the robustness of the developed machine learning model and therefore essential to achieve generalization in real-world situations.

## 4. Materials and Methods

### 4.1. Walnut Sample Preparation

In this experiment, a total of 192 walnut samples consisting of 10 varieties was collected from four provinces (see Figure S1 of supplementary material), i.e., Yunnan, Xinjiang, Shaanxi, and Hebei, which are known as the largest places of walnut production in China. The walnuts were harvested at commercial maturity and then transported to the laboratory at Zhejiang University, Hangzhou (120°09′ E, 30°14′ N), China. For analysis and spectral acquisition, the wholesome walnuts free from any abnormal features such as diseases, defects, and contaminations were collected. These walnut varieties had no obvious difference in appearance. Further details are presented in Table 4. These 10 varieties were simply labeled as No. 1–10. The number of samples collected from each variety was slightly different based on its availability, ranging from 16 to 20. Within each variety, around 2/3 samples were selected as the training set for model development, while the remaining ones served as the test set.

### 4.2. FT-MIR Spectroscopy Acquisition

The mid-infrared spectra of samples were acquired by a Jasco FT/IR-4100 spectrometer (Jasco International Co. Ltd., Tokyo, Japan) using the detector of DLATGSTGS and a ZnO crystal sampling accessory in transmission mode. Before collecting the spectrum, potassium bromide (KBr) was prepared by first drying in an oven at 105 °C for 4 h and then keeping in a vacuum drying dish. Each walnut shell sample was peeled, and about 5 g of sample was successively milled for 30 s using a grinder (FW100, Ty, instrument Co., Ltd., Shanghai, China). To prevent water absorption before spectra collection, the ground sample was packed in a dry sealed bag and stored in the vacuum desiccator. To effectively acquire FT-MIR spectra, 20 mg of walnut sample was homogenously mixed with the 980 mg KBr in a ratio of 1:49. A manual tableting machine produced by Jasco and matched with a Jasco FT/IR-4100 infrared spectrometer was used to compress the mixture at the same height for 1 min each time. When measuring, each sample was scanned 32 times at a resolution of 4 cm^−1^ in the range of 400–4000 cm^−1^. An average spectrum was taken as a representative of the sample. The data collection was performed at a constant temperature of 25 °C. The beginning and end of the original spectral data were eliminated to exclude the effect of the noise on the subsequent data analysis, leading to a smaller spectral range covering 700–3450 cm^−1^ for the modeling purpose.

### 4.3. Spectral Pre-Treatment

In this work, wavelet transform was used to smooth the spectral data. The basic idea behind wavelet denoising is that the wavelet transforms results in a sparse representation for many real-world signals. In other words, wavelet transform concentrates signal features in a few large-magnitude wavelet coefficients. Wavelet coefficients which are small in value are typically noise and we can “shrink” those coefficients or remove them without affecting the signal quality. After thresholding the coefficients, the data can be reconstructed using the inverse wavelet transform. The wavelet transform algorithm employs different basis functions and decomposition scales, leading to different denoising effects. In this case, the wavelet function Daubechies’ orthogonal wavelet basis Db3 and the decomposition scale 4 were used to denoise the spectral signal.

### 4.4. Optimal Spectral Variables Selection

We employed the genetic algorithm–partial least squares (GA-PLS) and uninformative variable elimination–successive projection algorithm (UVE-SPA) to select the optimal wavenumbers. As an adaptive heuristic search algorithm, GA can be applied when the dimension of the data space is too large for an exhaustive search [36]. It proceeds first by randomly generating an initial population of individuals, which should ideally cover the domain to explore. GA-based variable selection in the frame of PLS regression is thoroughly described in Leardi and González [37]. The basic principle of GA-PLS is to select candidate variables using GA and evaluate the selected subset using PLS. GA-PLS takes the minimum cross-validation root mean square error (RMSECV) or variance as the fitness and the PLS algorithm as the fitness evaluation function. Additionally, GA-PLS uses the weighted average of the frequency as the frequency of the final selected subset. In our case, the population size was set to 30, the crossover probability was set to 0.5, and the probability of mutation was set to 0.01 according to the previous studies performed by Leardi [37,38]. The GA-PLS tends to be more stable when the number of iterations increases and the number of selected variables decreases. Therefore, in this work, the number of GA-PLS iterations was set to 1000 to ensure the stability and accuracy of the results.

SPA is employed as a simple projection operation in a vector space to select subsets of variables with minimum collinearity. Compared to GA, SPA can provide more reproducible results [39]. Nevertheless, the SPA operation is time-expensive when the entire spectral range contains thousands of variables. UVE is used to eliminate the variables, which have no more informative variables for modeling than noise. Wu et al. [40] reported that the combination of UVE with SPA (UVE-SPA) could both reduce the calculation time and improve the model’s performance. In this work, the spectral variables selected by UVE were used as the input to feed into SPA with the range of the selected optimal wavenumbers setting to 5–30.

### 4.5. Principal Component Analysis

Principal component analysis (PCA) has been widely used for quantitative and qualitative analysis of spectral data. PCA linearly transforms the original data into new variables (i.e., scores and loadings). Each loading is a vector that provides information on the relative importance, or the weighting, of specific wavelengths relative to each other. Generally speaking, the first principal component (PC) contains the largest variance in the dataset and each following PC describes progressively less of the variance. In this sense, the first few PCs can be used to represent the original dataset, which greatly reduces the data dimension. In this work, PCA was used to visualize the distribution of data and hint at any possible clustering of the walnut powder samples based on different origins and varieties.

### 4.6. Machine Learning Algorithms

In this work, a wide range of classification modeling strategies were investigated including extreme learning machine (ELM), random forests (RF), back propagation neural network (BPNN), radial basis function (RBF) neural network, and partial least squares discrimination analysis (PLS-DA). The parameters and advantages of each classifier are summarized in Table S1 of the supplementary material. Classification models were developed separately based on the whole spectral region and the selected subsets of optimal variables. However, since it is quite time-consuming to build a BPNN model from the entire spectral range, this option is not considered.

ELM is a kind of single hidden layer feedforward neural network, which is a fast and simple classifier. In the ELM algorithm, only the number of hidden neurons need to be adjusted to obtain a unique and best solution, which is accomplished by comparing the effects of different neuron nodes. In this work, the number of neurons in the hidden layer was set from 1 to the size of the training set. The optimal number of neurons in the ELM model is determined by the minimum training error [41].

RF classifier is an integrated approach consisting of multiple decision trees that are independent of each other. The idea of RF is to build multiple decision trees and then merge them together to get a more accurate and stable prediction. As an ensemble method, RF has been proven to outperform a single decision tree because it reduces the over-fitting by averaging the result. Furthermore, RF is fast and tunable with relatively a small number of parameters [42].

PLS-DA is the discriminant analysis in the frame of PLS regression [43,44]. The discriminant analysis is conducted after the development of a PLS regression model built from spectral data and classes. The predicted values of the samples obtained from the regression model are not integers representing different categories, and therefore, thresholding is required. In the current work, the threshold was set to 0.5 [43]; that is, if the absolute value of the difference between the predicted value and the actual value was less than 0.5, the discrimination is acceptable and vice versa.

BPNN has a wide range of applications in regression and discriminant analysis [45,46]. It uses the error back propagation to modify the internal network weight after each training phase until the training error or the training phase of the network reaches the goal [47]. Herein, Matlab Neural Network Toolbox was used to train the BP neural network. The learning rate was set to 0.6. The number of iterations was 1000. The target deviation was 10^−5^. The other parameters follow the default settings. The discriminant threshold for BP neural network was set to 0.5, which is the same as PLS-DA.

RBF neural network is another artificial neural network, which is also widely used for various applications. RBF and BP neural network are non-linear multi-layer forward networks; RBF-NN usually has three layers: an input layer, a hidden layer with a non-linear RBF activation function, and an output layer. The output of the network is a linear combination of the radial basis functions of the input and neuron parameters [48]. All computations and machine-learning algorithms were performed with the aid of chemometric software Unscrambler^®^ 10.1 (CAMO AS, Oslo, Norway) and Matlab R 2014b (The Math Works, Natick, MA, USA).

## 5. Conclusions

The results obtained from this work highlight the effectiveness of FTIR combined with pattern recognition approaches in order to quickly and reliably identify the authenticity and provenance of walnut samples. BPNN modeling was successfully applied on the FT-MIR dataset, demonstrating that the spectroscopic fingerprint can serve as a fast screening platform for walnuts. Thus, these promising results should serve as an incentive for more research to be done on the development of a multifactorial approach combined with other techniques such as fluorescence spectroscopy, GC-MS, and NMR.

## Figures and Tables

**Table 1:** Modeling performances to classify four geographic origins on test set built from the full spectral range and subsets of the selected variable using different classifiers.

Classifier	Parameter	Yunnan	Xinjiang	Shaanxi	Hebei	Overall
ELM	62	84.62	70.00	100.00	52.63	74.24
RF	40	61.54	65.00	57.14	89.47	69.70
RBF	66	69.23	100.00	64.29	94.74	84.85
PLS-DA	12	69.23	50.00	71.43	84.21	68.18
UVE-SPA-ELM	56	61.54	90.00	71.43	94.74	81.82
UVE-SPA-RF	88	58.85	75.00	50.00	89.47	69.70
UVE-SPA-RBF	70	76.92	35.00	28.57	78.95	54.55
UVE-SPA-PLS-DA	6	53.85	90.00	100.00	89.47	84.85
UVE-SPA-BPNN	8	100.00	100.00	93.33	94.74	96.97
GA-PLS-ELM	108	69.23	85.00	71.43	78.95	77.27
GA-PLS-RF	60	58.85	75.00	50.00	89.47	69.70
GA-PLS-RBF	15	61.54	90.00	64.29	94.74	80.30
GA-PLS-PLS-DA	9	84.62	85.00	92.86	94.74	89.39
GA-PLS-BPNN	6	92.31	95.00	92.86	100.00	95.45

Note: Parameter: number of latent variables (LVs) for partial least squares–discrimination analysis (PLS-DA), number of forest trees for random forest (RF), number of nodes in the hidden layer for radial basis function (RBF), number of nodes for extreme learning machine (ELM), and number of neurons in the hidden layer for back propagation neural network (BPNN).

**Table 2:** Modeling performances to classify varieties within the same origin on a test set built from the full spectral range and subsets of the selected variable using different classifiers.

Origin	Variable Input	ELM	RF	RBF	PLS-DA	BPNN
Yunnan (No.1 No.2)	Full	84.62	84.62	92.31	84.62	-
	GA-PLS	92.31	84.62	92.31	92.31	100.00
	UVE-SPA	92.31	84.62	100.00	92.31	100.00
Xinjiang (No.3 No.4 No.5)	Full	70.00	65.00	90.00	70.00	-
	GA-PLS	90.00	65.00	90.00	65.00	94.74
	UVE-SPA	100.00	70.00	85.00	65.00	100.00
Shaanxi (No.6 No.7)	Full	85.71	92.31	100.00	100.00	-
	GA-PLS	100.00	100.00	100.00	100.00	100.00
	UVE-SPA	100.00	100.00	100.00	100.00	100.00
Hebei (No.8 No.9 No.10)	Full	73.68	68.42	68.42	78.95	-
	GA-PLS	78.95	73.68	73.68	78.95	94.74
	UVE-SPA	84.21	68.42	63.16	73.68	89.47

**Table 3:** Modeling performances to classify all 10 varieties on a test set built from the full spectral range and subsets of the selected variable using different classifiers.

	Classifier	ELM	RF	RBF	PLS-DA	BPNN
Variable Input						
Full		60.61	54.55	68.18	42.42	-
GA-PLS		68.18	53.03	71.21	60.61	87.88
UVE-SPA		66.67	48.48	60.61	51.52	83.33

**Table 4:** Details of the collected walnut samples and the characterization of each variety.

Province	Geographical Location	Variety	Characteristic	Sample Size	Data Partition (Training/Test Samples)
Yunnan	Southwest of China; 97°32′ ≈ 106°12′ E, 21°08′ ≈ 29°15′ N	No. 1: Yangbi Dapao	As the most planted variety in Yunnan, it is mainly distributed on the western slope of Cangshan Mountain in Yunnan, accounting for about 80% of Yangbi walnuts.	20	13/7
		No. 2: Yangbi Caoguo	It is mostly found in Meiji Village, West Town of Cangshan, Yunnan. The inner folds are well developed, and whole kernels can be collected.	19	13/6
Xinjiang	Northwest of China; 34°22′ ≈ 49°33′ E, 73°41′ ≈ 96°18′ N	No. 3: Hetian 185	It is the main walnut variety cultivated in Xinjiang, mostly found in southern Xinjiang.	19	13/6
		No. 4: Xinfeng	Grown at the altitude of 1700–2400 m, it is named after the skin, which is as thin as paper, and the whole kernel is easy to collect.	20	13/7
		No. 5: Xinxin 2	It is an early-maturing variety with the characteristics of high yield and good stability.	20	13/7
Shaanxi	Northwest of China; 105°29′ ≈ 111°15′ E, 31°42′ ≈ 39°35′ N	No. 6: Liao 4	As a crossbreed, this variety has strong adaptability, cold and drought tolerance, making it suitable for northern cultivation areas.	20	13/7
		No. 7: Xiangling	It is a mid-ripening variety, ideal for cultivation in thick and fertile soil conditions.	20	13/7
Hebei	Northern China; 113°04′ ≈ 119°53′ E, 36°01′ ≈ 42°37′ N	No.8: Qingxiang	It belongs to the late-maturing type, which was introduced from Japan.	16	10/6
		No.9: Liao 1	It is the main variety of walnut cultivated in Hebei.	18	12/6
		No.10: Liao 8	As one of the early-fruiting walnut varieties cultivated by hybridization, it gets mature in mid-September.	20	13/7

## Supplementary Materials

The following are available online. Figure S1. The mapping of the selected geographical regions in China. Figure S2. The raw mid-infrared spectrum of a randomly selected sample and the result after preprocessed by wavelet transform. Figure S3. The preprocessed mean spectra calculated from each variety. Figure S4. The CV of the number of variables included. Figure S5. Plot of 102 selected wavenumbers by GA-PLS. Figure S6. RMSE for selection by SPA (final number of selected variables:10, RMSE = 0.77742). Figure S7. Score plot for varieties from the same origin. PCA models were separately built from the samples within the same geographic origin, i.e., (a) Yunnan, (b) Xinjiang, (c) Shaanxi, (d) Hebei. Distinct separation between varieties can be observed in Yunnan (a) and Shaanxi (c). Figure S8. Score plot for all 10 varieties from four origins. Table S1. Parameters and advantages of the selected machine learning algorithms.
