# Supplementary material for: Authentication and Provenance of Walnut Combining Fourier Transform Mid-Infrared Spectroscopy with Machine Learning Algorithms
Source: Molecules. 2020 Oct 28;25(21):4987. doi: 10.3390/molecules25214987 (PMC7662659; doi:10.3390/molecules25214987)
Supplement: Supplementary file 1 [file molecules-25-04987-s001.pdf]

Authentication and Provenance of Walnut Combining Fourier Transform Mid-Infrared Spectroscopy with Machine Learning Algorithms

Hongyan Zhu [1], Jun-Li Xu [2],*

[1] College of Electronic Engineering, Guangxi Normal University, Guilin 541004, Guangxi, People's Republic of China; hyzhu-zju@foxmail.com
[2] UCD School of Biosystems and Food Engineering, University College of Dublin (UCD), Belfield, Dublin 4, Ireland; junli.xu@ucdconnect.ie.
* Correspondence: junli.xu@ucdconnect.ie

Table S1. Parameters and advantages of the selected machine learning algorithms.

ML algorithm	Parameter	Advantage
ELM	-Number of neurons in the hidden layer	-Fast<br>-Simple
RF	-Number of forest trees	-Reduce overfitting.,<br>-A small number of parameters
PLS-DA	-Threshold for discrimination.<br>-Number of latent variables.	-Simple,<br>-Easy interpretation
BPNN	-Learning rate<br>-Number of iterations<br>-Target deviation<br>-Threshold for discrimination<br>-Number of neurons in the hidden layer	-Straightforward to implement.<br>-Efficient at computing the gradient descent.
RBF-NN	-Number of nodes in the hidden layer	-Easy design<br>-Strong tolerance to input noise

Note: ELM: extreme learning machine; RF: random forests; PLS-DA: partial least squares discrimination analysis; BPNN: back propagation neural network; RBF-NN: radial basis function neural network.

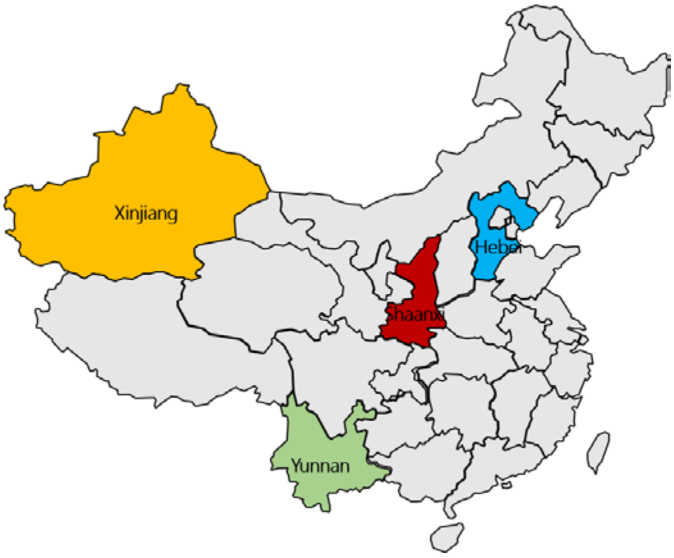

Figure S1. The mapping of the selected geographical regions in China.

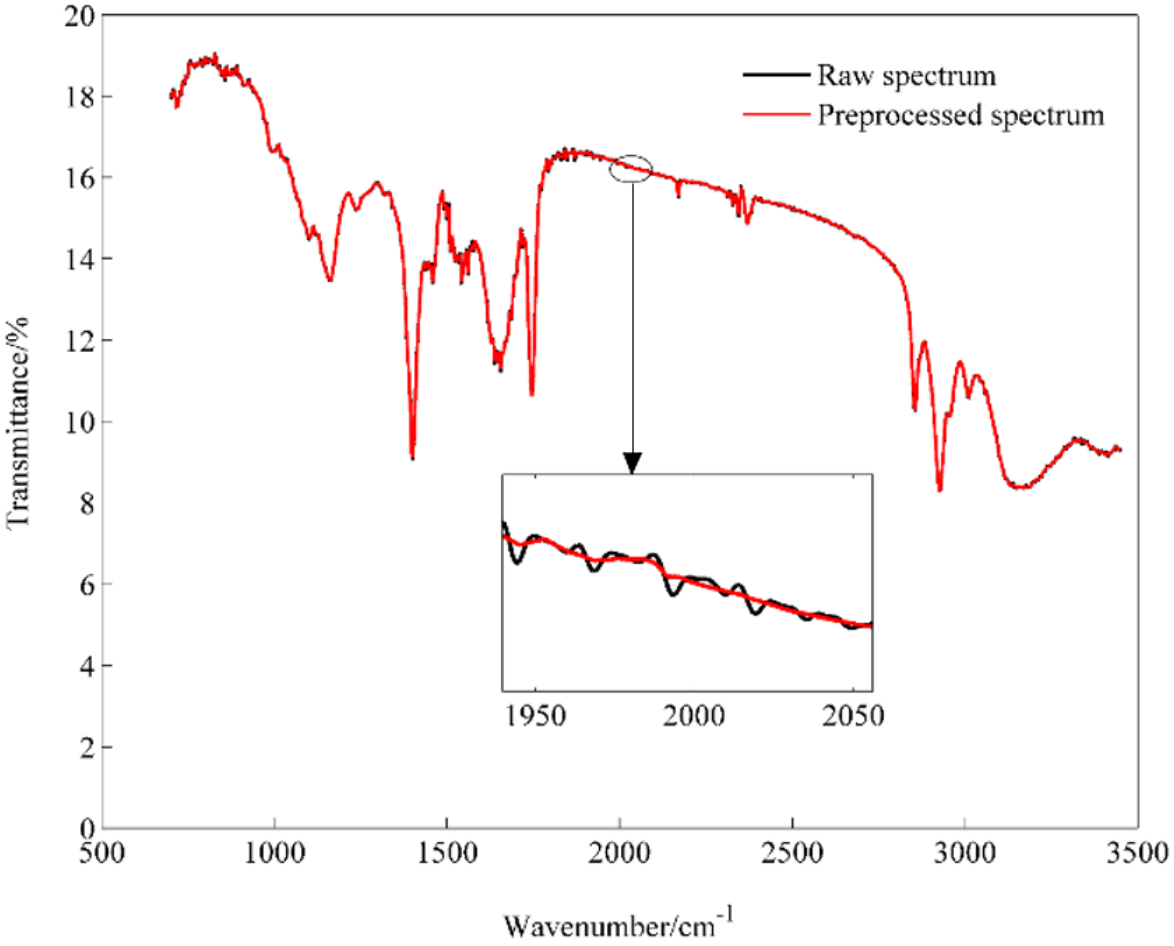

Figure S2. The raw mid-infrared spectrum of a randomly selected sample and the result after preprocessed by wavelet transform.

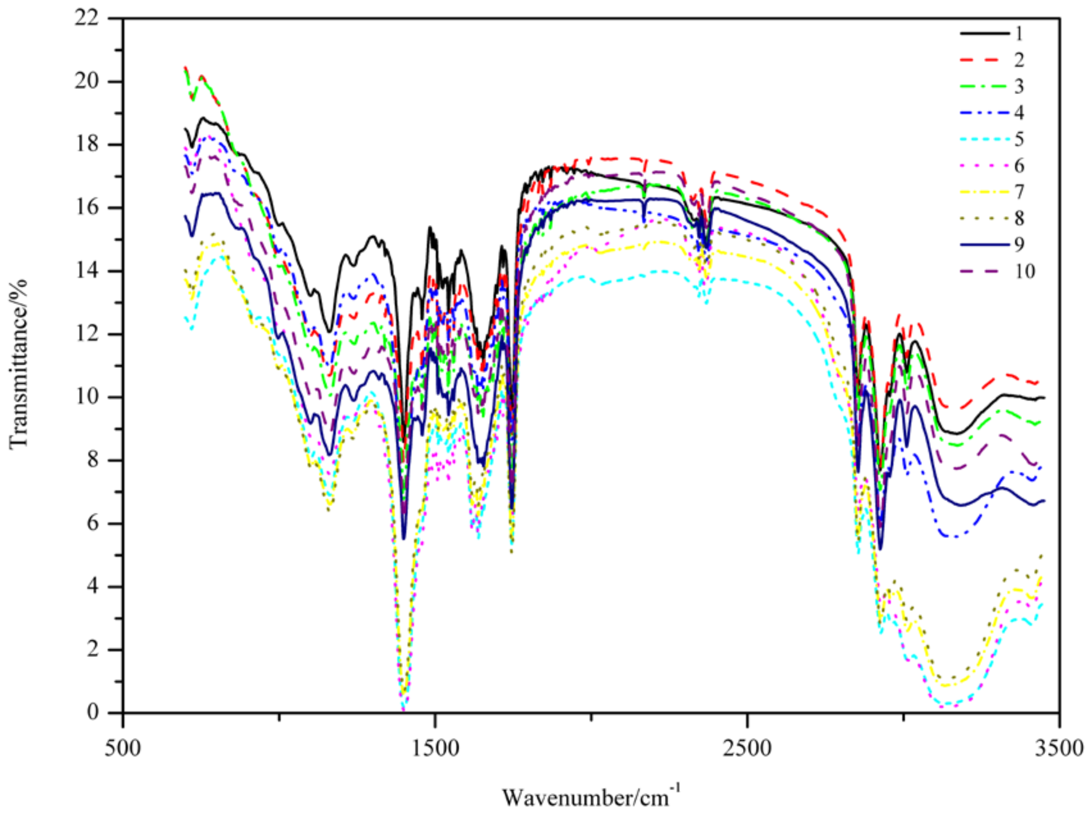

Figure S3. The preprocessed mean spectra calculated from each variety.

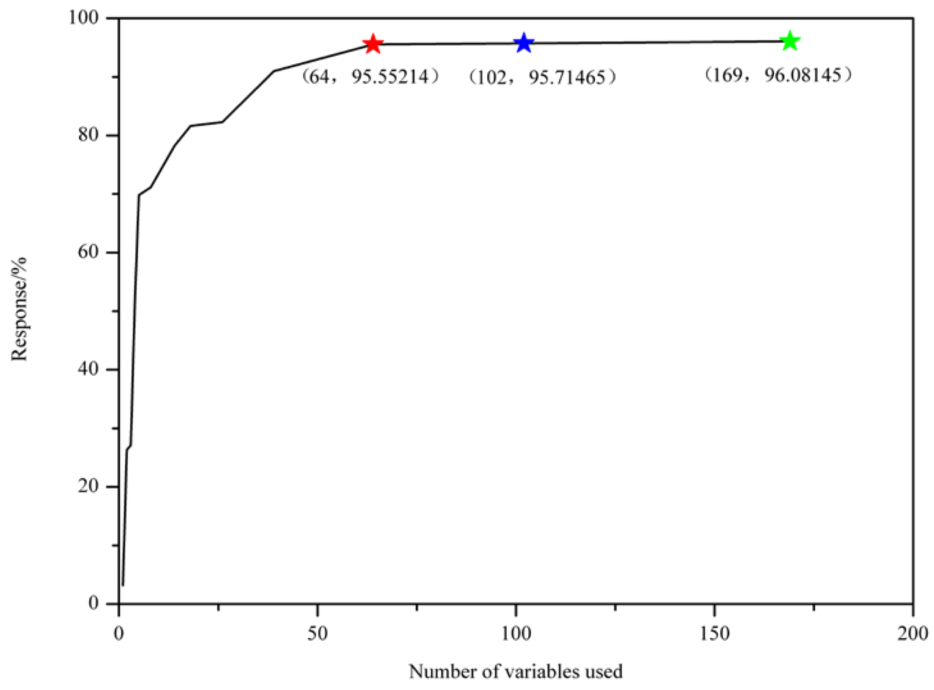

Figure S4. The CV of the number of variables included.

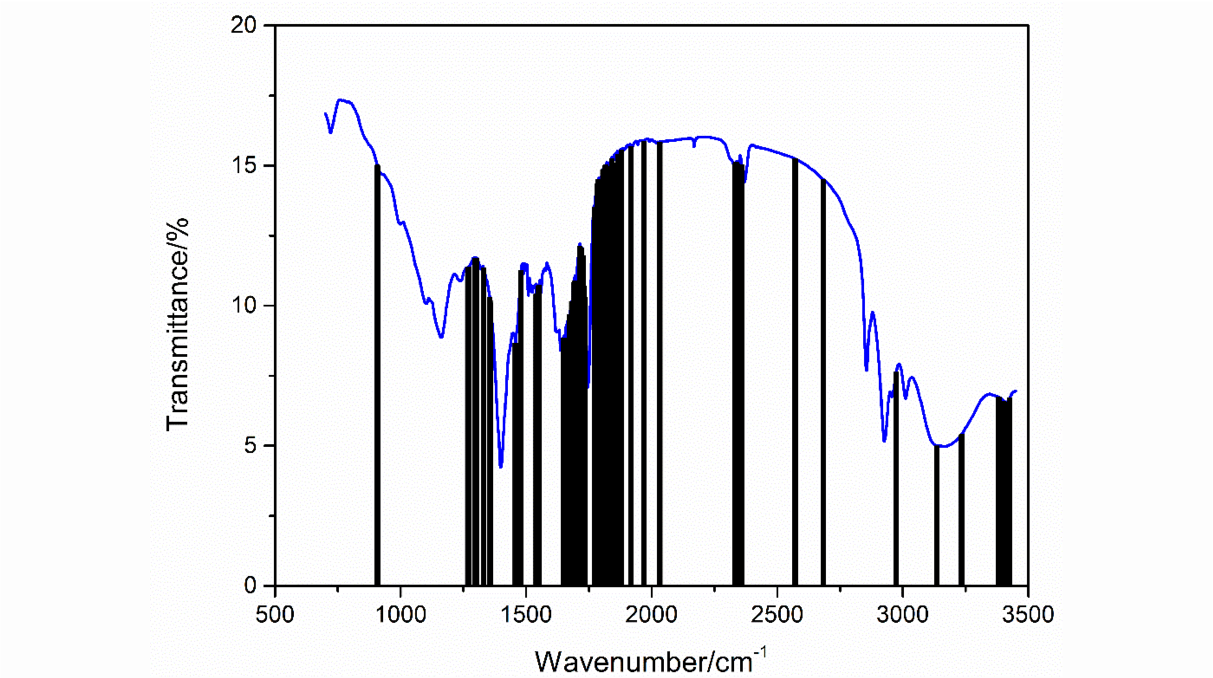

Figure S5. Plot of 102 selected wavenumbers by GA-PLS.

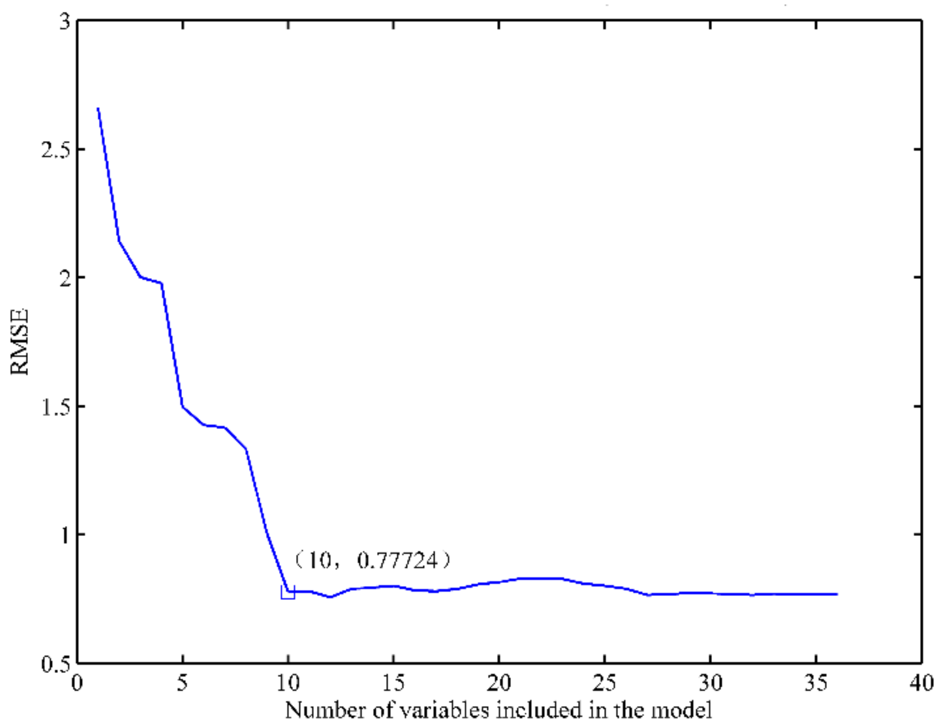

Figure S6. RMSE for selection by SPA (final number of selected variables:10, RMSE = 0.77742).

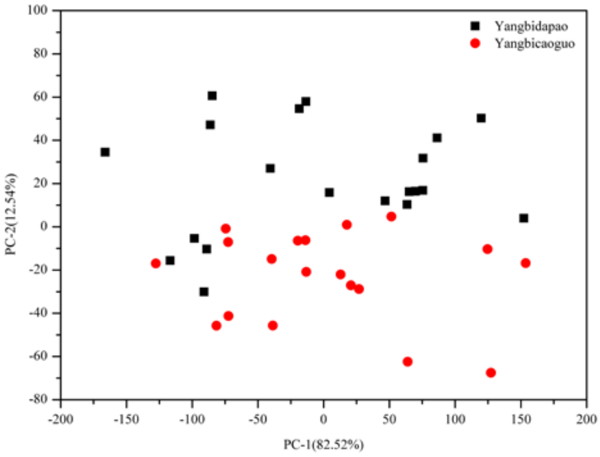

(A)Yunnan

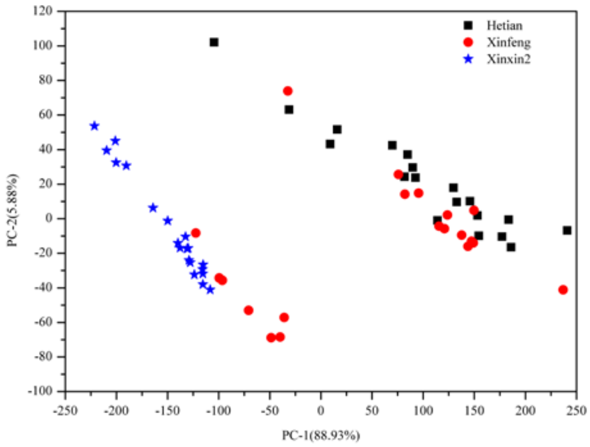

(b)Xinjiang

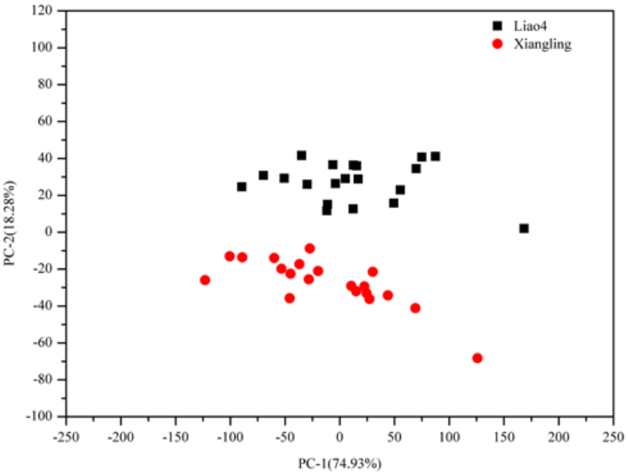

(c)Shaanxi

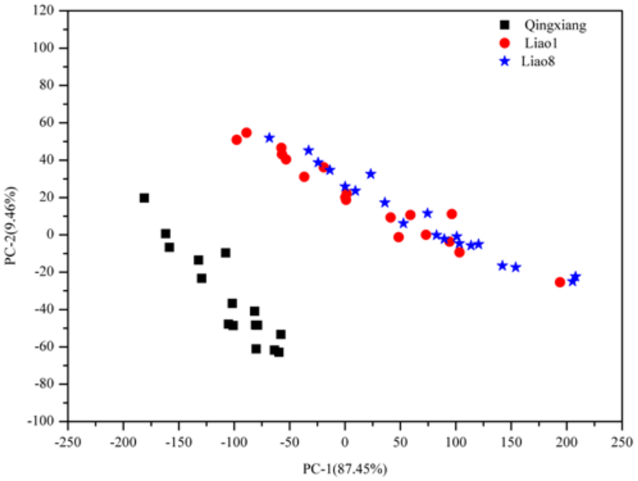

(d)Hebei

Figure S7. Score plot for varieties from the same origin. PCA models were separately built from the samples within the same geographic origin, i.e. (a) Yunnan, (b) Xinjiang, (c) Shaanxi, (d) Hebei. Distinct separation between varieties can be observed in Yunnan (a) and Shaanxi (c).

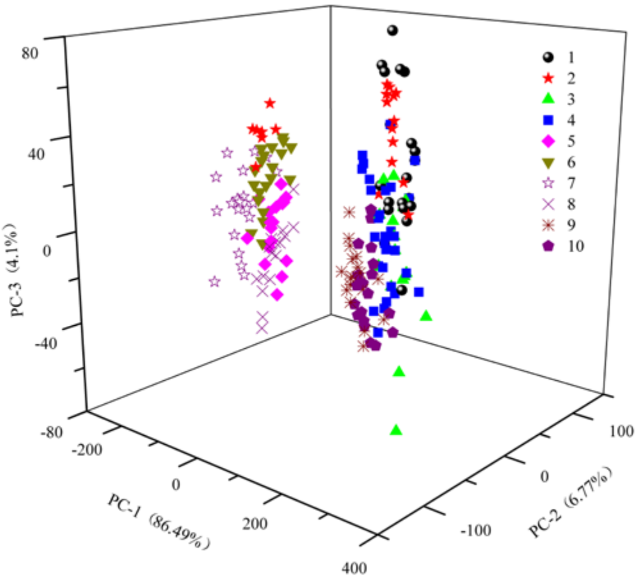

Figure S8. Score plot for all 10 varieties from four origins.

© 2020 by the authors. Licensee MDPI, Basel, Switzerland. This article is an open access article distributed under the terms and conditions of the Creative Commons Attribution (CC BY) license (http://creativecommons.org/licenses/by/4.0/).
